# The association of energy and macronutrient intake at breakfast and cardiovascular disease in Chinese adults: From a 14-year follow-up cohort study

**DOI:** 10.3389/fnut.2023.1093561

**Published:** 2023-03-20

**Authors:** Xiaoan Du, Ru Yang, Mengdi Ma, Songqing Ke, Jie Zheng, Xiaodong Tan

**Affiliations:** ^1^Wuhan Blood Center, Institute of Blood Transfusion of Hubei Province, Wuhan, China; ^2^School of Public Health, Wuhan University, Wuhan, China; ^3^School of Health and Nursing, Wuchang University of Technology, Wuhan, China

**Keywords:** carbohydrate, protein, fat, energy, breakfast, cardiovascular disease, CHNS

## Abstract

**Background:**

We aimed to examine the associations between energy and macronutrient intakes at breakfast and the incidence of cardiovascular events among Chinese adults.

**Methods:**

There were 12,937 participants from the China Health and Nutrition Survey who met the study criteria and completed six rounds of questionnaires in 1997, 2000, 2004, 2006, 2009, and 2011. Combined weighing methods with 24-h dietary recall were used to measure dietary intake throughout the day. Intakes of macronutrients at breakfast were calculated using energy provided by nutrients as a percentage of breakfast energy. We calculated hazard ratios using a multivariable Cox frailty model with random intercepts to account for household clustering.

**Results:**

During follow-up, we documented 453 (3.6 per 1,000 person-years) major cardiovascular events, 195 (1.5 per 1,000 person-years) myocardial infarctions, and 293 (2.3 per 1,000 person-years) strokes. In Chinese adults, more breakfast carbohydrates or less proteins intake was associated with the reduced risk of cardiovascular diseases. Especially for women, higher intake of breakfast carbohydrates was associated with a lower risk of major cardiovascular events (quintile 5 vs. quintile 1, HR 0.47 [95%CI 0.30–0.74]; *p*_trend_ = 0.0008) and stroke (quintile 5 vs. quintile 1, HR 0.48 [95%CI 0.26–0.88]; *p*_trend_ = 0.0006). Higher intake of breakfast proteins was associated with a higher risk of major cardiovascular events (quintile 5 vs. quintile 1, HR 1.77 [95%CI 1.12–2.79]; *p*_trend_ = 0.1162), myocardial infarction (quintile 5 vs. quintile 1, HR 2.49 [95%CI 1.21–5.11]; *p*_trend_ = 0.2641). There was a significant association between breakfast fat intake and cardiovascular diseases in the adult population, but less significant correlation was found in Chinese men or women. Breakfast fat intake was positively associated with the risk of major cardiovascular events (quintile 5 vs. quintile 1, HR 1.74 [95%CI 1.27–2.36]; *p*_trend_ = 0.0070), myocardial infarction (quintile 5 vs. quintile 1, HR 2.03 [95%CI 1.23–3.37]; *p*_trend_ = 0.0168), and stroke (quintile 5 vs. quintile 1, HR 1.64 [95%CI 1.12–2.41]; *p*_trend_ = 0.0732). There was a significant reduction in major cardiovascular events and stroke when breakfast energy intake was moderated, even if the independence of skipping breakfast.

**Conclusion:**

High carbohydrate intake and low protein and fat intake at breakfast may contribute to cardiovascular health while maintaining a moderate energy intake.

## 1. Introduction

In 2018, cardiovascular disease (CVD) mortality in China remained at the top of the disease spectrum (1), accounting for an average of two out of every five deaths. Stroke, as one of the major CVD, has become the leading cause of death among adults in China (2). According to the “China Cardiovascular Health and Disease Report 2020” (1), approximately 12 million people in China are estimated to have strokes, and 11.39 million have coronary heart disease such as myocardial infarction (MI). CVD has rapidly transformed from a disease in developed countries to a global disease with increasing prevalence and incidence in low-income countries. The prevailing risk factors for cardiovascular disease, namely hypertension, and diabetes mellitus, along with the unhealthy poor-quality diet, would contribute to this complex transition (3).

The presence of poor eating habits or dietary factors has been validated to be associated with a range of chronic diseases (4). As advocated by “2021 Dietary Guidance to Improve Cardiovascular Health,” a low-quality diet is strongly associated with cardiovascular morbidity and mortality (5). Equally, meal timing as well as daily nutrient intake regulates cardiovascular risk. There was evidence that the timing of food consumption may alter the circadian rhythm of metabolism, which in turn affects the biological clock (6, 7). According to studies, late-lunch eating (8) and late-night eating (9, 10) were related to a higher risk of cardiometabolic health. Similarly, breakfast skipping or irregular breakfast eating habits were associated with a greater risk of CVD (11–13), though a recent review on the association of breakfast skipping with cardiovascular disease has drawn a controversial conclusion (14). However, with the accelerated pace of life and the deep-rooted concept of weight control and weight loss, breakfast skipping, as a part of the strategies proposed for reducing energy intake, is becoming more prevalent (15, 16). The “Survey Report on the Status of Chinese Residents’ Breakfast Diet” issued by the Chinese Nutrition Society has shown that more than 30% of Chinese people cannot eat breakfast every day. Noticeably, it cannot be assumed that people who eat breakfast are necessarily healthier. The basic principle of a healthy breakfast is to try to ensure that the variety of food is as diverse as possible and nutrient intake at breakfast is reasonable.

Studies have investigated the beneficial effects of regular eating breakfast on risk factors of cardiovascular disease (11, 17), but subsequent results remain inconsistent, possibly because of different breakfast compositions. A series of studies have supported that total energy consumption at breakfast reduced weight gain and CVD risk factors, such as elevated serum low-density lipoprotein cholesterol (18, 19). An animal study also has made a similar conclusion that high energy intake at breakfast has a favorable regulation of blood lipids (20). There was evidence, both from a randomized controlled trial (21) and a prospective observational study (22), showing that a reduction in dietary saturated fatty acids (SFA) reduces the risk of CVD. While few studies have been conducted that assess whether fat intake at breakfast has any effect on CVD, researchers found a reduction in the risk of intracerebral hemorrhage associated with higher saturated or monounsaturated fat consumption at breakfast in Japanese men (23). Correspondingly, greater protein intake at breakfast could reduce body weight (18, 24), and was inversely associated with systolic and diastolic pressure and positively associated with high-density lipoprotein cholesterol (25), which may have beneficial effects on cardiovascular health. Protein intake from plant sources was associated with a lower risk of cardiovascular disease mortality (26), whereas protein intake from animal sources with a higher risk of cardiovascular health (27). Given to different animal sources, many studies have provided much evidence that red meats, such as poultry and beef, were associated with a range of adverse cardiovascular health (28–30). Furthermore, processed meat that contain high amounts of sodium have been linked with a higher risk of CVD incidence (30).

In most nutritional guidelines, there is a lack of recommendations regarding eating habits (timing, quantity, energy content, and frequency) in adults (31, 32). During the past two decades, the overall energy intake in Chinese adults has shown a decreasing trend, and the dietary structure significantly changed (33). However, a longitudinal study has not been conducted to date to examine the relationship between macronutrient intake at breakfast and the risk of cardiovascular disease. In this study, our primary aim was to assess the association of energy and macronutrient (carbohydrate, protein, and fat) intake at breakfast with cardiovascular disease events among Chinses adults. The secondary aim was to investigate the effect of energy and macronutrient intake at breakfast on cardiovascular diseases in participants with the presence of hypertension.

## 2. Materials and methods

### 2.1. Study participants

**Figure 1:**
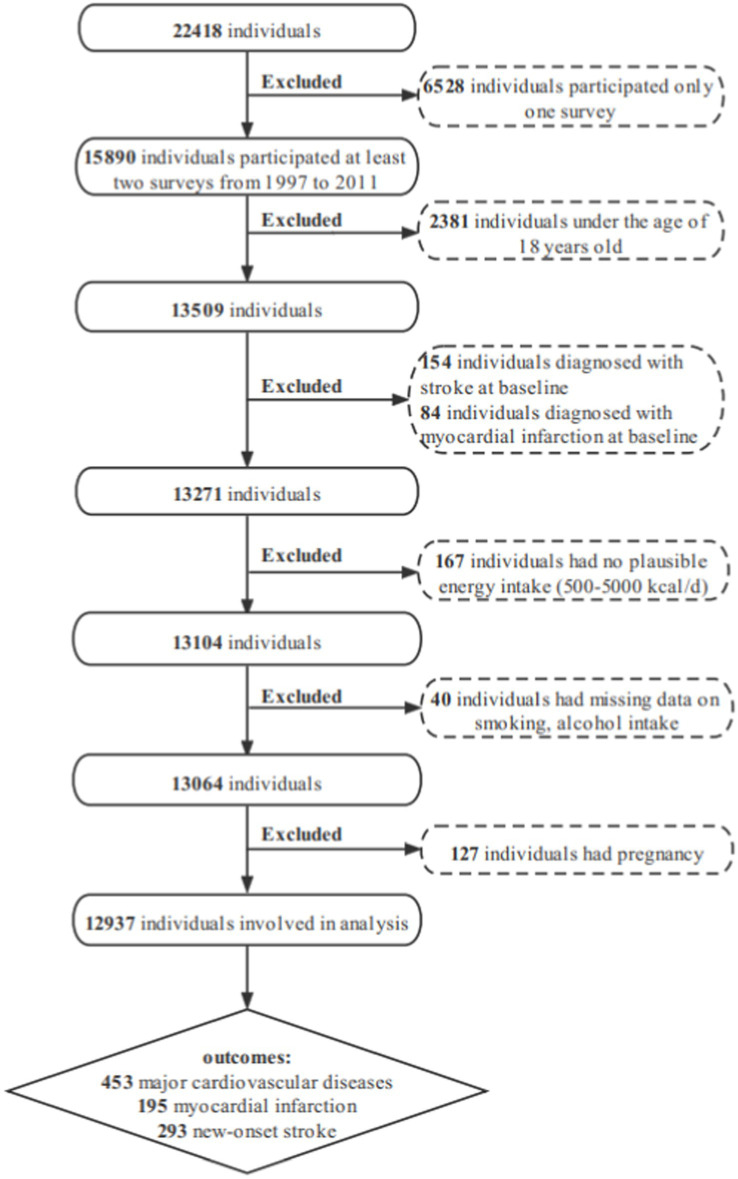
Flow chart of sample selection.

In this study, data were derived from a stratified, multistage prospective survey of the China Health and Nutrition Survey (CHNS), a cohort study. More details of the sampling process and design used in CHNS have previously been published elsewhere (34). The food codes used before 1997 in CHNS did not match the published China Food Composition tables (35), and the 2015 dietary survey data have not yet been published. In brief, data on 22,418 participants were collected in nine provinces from 1997, 2000, 2004, 2006, 2009, and 2011, including Liaoning, Heilongjiang, Jiangsu, Shandong, Henan, Hubei, Hunan, Guangxi and Guizhou. The following participants were excluded from the present analysis: 6528 participants participated in only one wave; 2,381 individuals aged less than 18 years old at baseline; 238 individuals had a myocardial infarction, or stroke at baseline; 167 individuals had implausible energy intake (<500 or > 5,000 kcal per day); 40 individuals had missing data on smoking, alcohol intake; 127 individuals had a pregnancy. A total of 12,937 individuals were involved in this analysis (Figure 1).

### 2.2. Dietary assessment

Dietary intake was evaluated at the household and individual level using the household food stock method combined with three consecutive 24-h recalls (36, 37), which means selected for 3 consecutive days from Monday to Sunday according to the randomization principle. Investigators were required to receive standardized training on dietary survey techniques and proper food estimation methods before conducting the household dietary survey. The classification of food categories is based on the food codes recorded in the CHNS database. The food codes in the CHNS database have used two different systems of food coding. In particular, food codes from the Chinese Food Composition Table (1991) were used in 1997 and 2000 to calculate individual daily intake of select nutrients for each food item. From 2004 to 2011, surveys were based on the 2002 edition of the Chinese Food Composition Table with a supplement of the 2004 edition. Noteworthy, different food sources under the same category cannot be directly summed up. The differences in energy and nutrients provided by different food sources should be noted when estimating food consumption, to ensure the accuracy of meal data. A double labeled water method has been validated in this survey to determine total energy intake from combined dietary intake (38). Calculation of the proportion of energy derived from nutrients (carbohydrate, protein, and fat) at breakfast is as follows: for carbohydrate, [carbohydrate intake (g/day) × 4]/breakfast energy intake (kcal/day) × 100; for fat, [fat intake (g/day) × 9]/breakfast energy intake (kcal/day) × 100; for protein, [protein intake (g/day) × 4]/breakfast energy intake (kcal/day) × 100. Additionally, a cumulative method on the averages of energy and macronutrients was applied to provide more weight to individuals’ baseline diet (39), and represented long-term habitual dietary intakes.

### 2.3. Health outcomes

Myocardial infarction and stroke onset were obtained by face-to-face or telephone interview according to the study participant’s self-report of previous diagnosis and treatment, and only if that diagnosis had been made by a professional physician. When asked, “Has there been a myocardial infarction diagnosed in the past year by a doctor?,” a positive answer indicated a myocardial infarction; stroke was defined as a positive answer to the question “Have you been diagnosed with a stroke or transient ischemic attack by a doctor?” Major cardiovascular events included myocardial infarction and stroke. The follow-up period was calculated as the time from their initial year of survey participation to either an expected outcome event or lost to follow-up or the censoring date. As to avoid temporal bias, for example, if an individual participated the first wave in 1997, for cardiovascular outcomes during the 1997–2000 time period, the follow-up period was calculated as the following: [2000–1997 year]/2. Here we summarized the formula as the following:

The follow-up period = [(Y_n-1_-Y_1_) + (Y_n_-Y_n-1_)/2], n ≥ 2.

The six rounds of survey were conducted in 1997, 2000, 2004, 2006, 2009 and 2011. In this formula, n indicates the number of times participated in the survey, Y_n_ indicates the survey year of n wave, Y_n-1_ indicates the survey year of n-1 wave, Y_1_ indicates the year of the first participation in the survey.

### 2.4. Confounding variables

Sociodemographic characteristics including age (years), gender (men/women), ethnicity (Han/ethnic minorities), living area (urban/rural), education level (primary school or lower/Lower middle school/Middle school or above), region (Northeast/East Coast/Central/West) and annual household income (Yuan) were assessed in the questionnaires. This study was conducted to determine the presence of hypertension according to hypertension control guidelines (40). Hypertension was determined when one of the following conditions was met: (1) those who self-reported history of diagnosis/treatment of hypertension; (2) those who had taken antihypertensive drugs; (3) systolic blood pressure (SBP) ≥ 140 mmHg or diastolic blood pressure (DBP) ≥ 90 mmHg at physical measurement. SBP/DBP were checked by trained staff. Diabetes was determined when one of the following conditions was met: (1) those who self-reported having diabetes in the questionnaire; (2) those who had a positive answer to the question “If you had been diagnosed with diabetes, had you used any of the following treatments (i.e., oral hypoglycemic drugs, or Chinese medicine, or insulin injection)?” Body mass index (BMI) was calculated by the formula “BMI = weight (kg)/height (m^2^).” According to the Chinese BMI reference standard, BMI < 18.5 kg/m^2^ is defined as underweight, 18.5 ≤ BMI < 24 kg/m^2^ as healthy weight, 24 ≤ BMI < 28 kg/m^2^ as overweight, and BMI ≥28 kg/m^2^ as obesity. Physical activity level was assessed based on time spent per week in different occupational, domestic, transportation and leisure activities using a validated questionnaire that calculates the Metabolic Equivalent of Task (MET) for each activity according to the Physical Activity Compendium (41, 42). Smoking status was categorized as a non-smoker, ex-smoker, and current smoker. Alcohol intake was classified on the basis of frequency of alcohol consumption (non-drinker, < 3 times per week and ≥ 3 times per week). Dietary measurements included dietary fiber (g/day), grains (g/day), vegetables (g/day), total energy intake (kcal/day), SFA (g/day); breakfast skipping (yes/no).

### 2.5. Statistical analysis

Continuous variables are summarized as means and stand deviations (SDs) and categorical variables as percentages. We evaluated the proportional hazards assumption using the Schoenfeld residual method combined with visual inspection of log–log plots, which were consistent with proportional hazards. Hazard ratios (HRs) of three cardiovascular outcomes (major cardiovascular events, MI, and stroke) attributed to energy intake including breakfast energy, the proportion of energy from macronutrients at breakfast were calculated using a multivariable Cox frailty model with random intercepts to account for household clustering. For the overall analysis, participants were categorized into quintiles of energy intake at breakfast and nutrient intake (carbohydrate, fat, and protein) at breakfast based on the percentage of energy provided by nutrients. The lowest quintile category was used as the reference group. Minimally adjusted models were adjusted for age, sex, and household clustering as a random effect. Maximally adjusted models were further adjusted for an urban or rural location, education level, income, physical activity, smoking status, alcohol intake, BMI, history of hypertension, diabetes, total energy intake, saturated fatty acid (SFA), grains, vegetables, and dietary fiber. In addition, we used restricted cubic splines with four knots (at the 5th, 35th, 65th, and 95th) to investigate the shape of the association between breakfast nutrient intakes and outcomes. We further built two sets of predicted isocaloric models to estimate the effect of isocaloric replacement (as 5% of energy) of carbohydrates at breakfast with protein and fat intake at breakfast when total energy intake at breakfast was constant (43), simultaneously including energy intake at breakfast and percentages of energy from carbohydrate and other specific macronutrients at breakfast (continuous), as well as other potential confounders. Interactions were explored between energy and macronutrient intake at breakfast and the presence of hypertension. We introduced a cross-product interaction term in the multivariable model to assess the significance of the interaction and to examine whether the effect of energy and macronutrient intake at breakfast on events differed in participants with or without hypertension. The fully adjusted model was subjected to sensitivity analysis by the addition of skipping breakfast. The significance threshold for all comparisons was set at 5% for all *p* values. All statistical analyses were performed with R software, version 4.1. Spline curves were generated with the *“rms”* package.

## 3. Results

### 3.1. Participants’ characteristics at baseline and health outcomes

Between 1997 and 2011, a total of 12,937 participants aged 18–93 years old (mean ± SD, 43.2 ± 14.9) at baseline were included and analyzed in this study, of which 6,286 (48.6%) were males. The median follow-up of 11 years (IQR 7–14 years), during which data regarding major cardiovascular events were available for 453 (3.6 per 1,000 person-years) participants, all myocardial infarctions were available for 195 (1.5 per 1,000 person-years) participants and new-onset strokers were available for 293 (2.3 per 1,000 person-years) participants.

Demographic characteristics of participants and data on dietary intake at baseline according to quintiles of energy intake at breakfast are presented in Table 1. Compared with those in quintiles 1–4, participants in the fifth quintile who consumed more energy at breakfast were more likely to be men, poorly educated, those with high physical activity levels, and those who lived in rural areas and were from regions of central China. Participants who were to be no smokers or alcohol drinkers at baseline were more likely to appear in the fifth quintile. Breakfast energy intake was associated with higher intakes of total energy and carbohydrate and lower intakes of protein and fat at baseline.

**Table 1:** Baseline characteristics according to quintiles of energy intake at breakfast: CHNS, 1997–2011.

Characteristics	Quintile 1 (*n* = 2,588) < 268.2 kcal/d	Quintile 2 (*n* = 2,587) 268.2–359.0 kcal/d	Quintile 3 (*n* = 2,587) 359.0–443.3 kcal/d	Quintile 4 (*n* = 2,587) 443.3–562.6 kcal/d	Quintile 5 (*n* = 2,588) > 562.6 kcal/d	*p* value
Age, years	43.16 ± 15.10	42.96 ± 14.64	43.75 ± 14.90	43.09 ± 14.83	43.29 ± 15.01	0.373
Male, %	1,090 (42.1)	1,022 (39.5)	1,179 (45.6)	1,400 (54.1)	1,595 (61.6)	< 0.001
Han, %	2,208 (85.3)	2,323 (89.8)	2,327 (89.9)	2,279 (88.1)	2,226 (86.0)	< 0.001
Urban residence, %	875 (33.8)	869 (33.6)	960 (37.1)	927 (35.8)	881 (34.0)	0.03
Education						
Primary school or lower	1,168 (45.1)	1,194 (46.2)	1,152 (44.5)	1,182 (45.7)	1,209 (46.7)	< 0.001
Lower middle school	724 (28.0)	747 (28.9)	813 (31.4)	834 (32.2)	892 (34.5)	
Middle school or above	696 (26.9)	646 (25.0)	622 (24.0)	571 (22.1)	487 (18.8)	
Region						
Northeast	435 (16.8)	473 (18.3)	621 (24.0)	644 (24.9)	497 (19.2)	< 0.001
East coast	452 (17.5)	575 (22.2)	595 (23.0)	636 (24.6)	643 (24.8)	
Central	602 (23.3)	997 (38.5)	910 (35.2)	868 (33.6)	896 (34.6)	
West	1,099 (42.5)	542 (21.0)	461 (17.8)	439 (17.0)	552 (21.3)	
Family household income^a^, yuan	5777.20 ± 6973.13	6060.39 ± 8012.01	6502.67 ± 7622.00	6278.35 ± 7192.21	6048.89 ± 7136.41	0.007
Physical activity, MET-h/w	161.61 ± 78.51	165.15 ± 92.30	179.85 ± 97.44	188.26 ± 100.74	199.15 ± 102.48	< 0.001
BMI, kg/m^2^						
< 18.5	171 (6.6)	189 (7.3)	151 (5.8)	115 (4.4)	111 (4.3)	< 0.001
18.5 to 23.9	1,621 (62.6)	1715 (66.3)	1,699 (65.7)	1726 (66.7)	1732 (66.9)	
≥ 24	796 (30.8)	683 (26.4)	737 (28.5)	746 (28.8)	745 (28.8)	
SBP, mmHg	119.84 ± 16.15	120.28 ± 17.04	120.98 ± 16.38	120.98 ± 16.23	119.96 ± 16.41	0.023
DBP, mmHg	77.82 ± 10.04	77.78 ± 10.28	78.71 ± 10.13	78.52 ± 10.51	78.09 ± 9.96	0.002
History of hypertension, %	347 (13.4)	126 (4.9)	123 (4.8)	104 (4.0)	128 (4.9)	< 0.001
History of diabetes, %	95 (3.7)	24 (0.9)	21 (0.8)	12 (0.5)	18 (0.7)	< 0.001
Smoking status						
Never	1941 (75.0)	1962 (75.8)	1868 (72.2)	1723 (66.6)	1,610 (62.2)	< 0.001
Former	49 (1.9)	40 (1.5)	31 (1.2)	35 (1.4)	48 (1.9)	
Current	598 (23.1)	585 (22.6)	688 (26.6)	829 (32.0)	930 (35.9)	
Alcohol intake						
Never	1880 (72.6)	1858 (71.8)	1739 (67.2)	1,670 (64.6)	1,672 (64.6)	< 0.001
< 3 times per week	390 (15.1)	415 (16.0)	466 (18.0)	499 (19.3)	499 (19.3)	
≥ 3 times per week	318 (12.3)	314 (12.1)	382 (14.8)	418 (16.2)	417 (16.1)	
Dietary intake						
Total energy, kcal/d	2032.66 ± 631.08	2111.11 ± 567.66	2217.68 ± 563.96	2373.77 ± 610.89	2608.64 ± 668.76	< 0.001
Carbohydrate, % energy/d	57.47 ± 13.69	58.54 ± 12.12	60.31 ± 11.81	61.33 ± 11.85	64.07 ± 11.38	< 0.001
Protein, % energy/d	12.60 ± 2.96	12.47 ± 2.71	12.18 ± 2.64	12.11 ± 2.60	11.87 ± 2.38	< 0.001
Fat, % energy/d	29.25 ± 12.81	28.33 ± 11.70	26.76 ± 11.13	25.81 ± 11.14	23.56 ± 11.07	< 0.001
Protein at breakfast, % breakfast energy^b^	13.11 ± 9.67	12.04 ± 3.66	11.97 ± 3.22	11.57 ± 3.36	10.61 ± 3.97	< 0.001
Fat at breakfast, % breakfast energy	12.60 ± 15.45	10.44 ± 8.76	11.21 ± 8.56	10.88 ± 8.70	10.93 ± 8.97	< 0.001
Carbohydrates at breakfast, % breakfast energy	59.31 ± 31.86	77.57 ± 11.08	76.91 ± 10.23	77.70 ± 11.15	78.59 ± 11.04	< 0.001
SFA, g/d	22.99 ± 15.05	23.78 ± 17.36	23.92 ± 18.46	25.01 ± 20.37	26.83 ± 23.12	< 0.001
Grains, g/d	379.48 ± 172.11	406.49 ± 140.37	451.11 ± 151.34	493.58 ± 164.65	585.32 ± 211.61	< 0.001
Vegetables, g/d	279.40 ± 152.26	300.70 ± 158.67	306.42 ± 160.03	319.22 ± 166.90	331.13 ± 169.36	< 0.001
Dietary fiber, g/d	9.12 ± 5.89	9.06 ± 3.94	9.98 ± 4.31	10.70 ± 4.79	11.81 ± 6.81	< 0.001

An ANOVA was used to test the differences in continuous variables across quintiles of energy intake at breakfast, and data were depicted by means ± SDs; a chi-square test was used for categorical variables, and data were depicted by frequencies (%). ^a^ Family household income was inflated to 2015. ^b^ refers to the percentage of breakfast energy intake from macronutrients at breakfast.

### 3.2. Association between health outcomes and energy and macronutrients intake

The hazard ratios (HRs) and 95% confidence intervals (CIs) for the association between breakfast energy and macronutrient intake and health outcomes are shown in Table 2. Higher percentage energy from carbohydrate intake at breakfast was associated with a lower risk of major cardiovascular events (quintile 5 vs. quintile 1, HR 0.60 [95%CI 0.44–0.81]; *p*_trend_ < 0.0001), new-onset stroke (quintile 5 vs. quintile 1, HR 0.58 [95%CI 0.40–0.86]; *p*_trend_ = 0.0121). In comparisons between quintile 5 and quintile 1, percentage energy from protein intake at breakfast was associated with a higher risk of major cardiovascular events (quintile 5 vs. quintile 1, HR 1.72 [95%CI 1.25–2.36]; *p*_trend_ = 0.0024), myocardial infarction (quintile 5 vs. quintile 1, HR 1.81 [95%CI 1.09–3.01]; *p*_trend_ = 0.0058), and new-onset stroke (quintile 5 vs. quintile 1, HR 1.53 [95%CI 1.04–2.25]; *p*_trend_ = 0.1606), after multivariable adjustment for covariates. Similarly, percentage energy from the fat intake at breakfast was positively associated with risks of major cardiovascular events (quintile 5 vs. quintile 1, HR 1.74 [95%CI 1.27–2.36]; *p*_trend_ = 0.0070), myocardial infarction (quintile 5 vs. quintile 1, HR 2.03 [95%CI 1.23–3.37]; *p*_trend_ = 0.0168), and new-onset stroke (quintile 5 vs. quintile 1, HR 1.64 [95%CI 1.12–2.41]; *p*_trend_ = 0.0732).

**Table 2:** Association between energy and percentage energy from macronutrients at breakfast and health outcomes.

	Incidence (per 1,000 person-years; 95% CI)					Hazard ratio (95% CI)				*p*_trend_
	Quintile 1	Quintile 2	Quintile 3	Quintile 4	Quintile 5	Quintile 2 vs. Quintile 1	Quintile 3 vs. Quintile 1	Quintile 4 vs. Quintile 1	Quintile 5 vs. Quintile 1	
The energy at breakfast (kcal/d)										
The range of value	< 268.2	268.2–359.0	359.0–443.3	443.3–562.6	> 562.6					
Major cardiovascular events	5.6	3.7	3.3	2.6	3.1	0.95	0.91	0.71	0.86	0.1364
	(4.6–6.6)	(3.0–4.4)	(2.6–4.0)	(2.0–3.2)	(2.4–3.8)	(0.72–1.26)	(0.68–1.22)	(0.52–0.98)	(0.62–1.20)	
Myocardial infarction	1.8	1.9	1.7	1.2	1.1	1.51	1.44	1.03	0.95	0.506
	(1.2–2.3)	(1.4–2.5)	(1.2–2.2)	(0.8–1.6)	(0.7–1.5)	(0.98–2.33)	(0.92–2.25)	(0.63–1.67)	(0.55–1.63)	
Stroke	4.1	2	1.9	1.6	2.2	0.73	0.73	0.61	0.81	0.16
	(3.2–4.9)	(1.5–2.6)	(1.4–2.5)	(1.1–2.1)	(1.6–2.8)	(0.51–1.04)	(0.51–1.06)	(0.41–0.91)	(0.55–1.21)	
Energy from carbohydrates (% breakfast energy)										
The range of value	< 66.6	66.6–75.5	75.5–81.3	81.3–86.3	> 86.3					
Major cardiovascular events	6.7	3.2	2.9	2.8	3.1	0.7	0.73	0.61	0.6	< 0.0001
	(5.5–7.8)	(2.5–3.9)	(2.3–3.6)	(2.2–3.4)	(2.5–3.8)	(0.52–0.94)	(0.54–0.99)	(0.45–0.83)	(0.44–0.81)	
Myocardial infarction	2.6	1.6	1.4	1.1	1.2	0.92	0.87	0.65	0.69	0.0529
	(1.9–3.3)	(1.1–2.1)	(0.9–1.8)	(0.7–1.5)	(0.8–1.6)	(0.60–1.40)	(0.56–1.35)	(0.40–1.04)	(0.43–1.11)	
Stroke	4.5	1.8	1.7	1.9	2.2	0.59	0.66	0.62	0.58	0.0121
	(3.6–5.4)	(1.2–2.3)	(1.2–2.2)	(1.4–2.5)	(1.6–2.7)	(0.40–0.87)	(0.45–0.97)	(0.43–0.89)	(0.40–0.86)	
Energy from protein (% breakfast energy)										
The range of value	< 8.9	8.9–11.3	11.3–12.6	12.6–14.3	> 14.3					
Major cardiovascular events	3	3.5	3.1	3.1	5.6	1.26	1.18	1.23	1.72	0.0024
	(2.3–3.7)	(2.7–4.2)	(2.5–3.8)	(2.4–3.7)	(4.6–6.6)	(0.91–1.75)	(0.85–1.64)	(0.88–1.73)	(1.25–2.36)	
Myocardial infarction	1.1	1.2	1.3	1.8	2.4	1.21	1.29	1.81	1.81	0.0058
	(0.7–1.5)	(0.8–1.7)	(0.9–1.7)	(1.3–2.3)	(1.7–3.0)	(0.71–2.06)	(0.76–2.18)	(1.09–3.00)	(1.09–3.01)	
Stroke	2.5	2.5	2.1	1.5	3.6	1.24	1.07	0.88	1.53	0.1606
	(1.9–3.1)	(1.9–3.1)	(1.5–2.6)	(1.1–2.0)	(2.8–4.3)	(0.84–1.82)	(0.72–1.59)	(0.57–1.35)	(1.04–2.25)	
Energy from fat (% breakfast energy)										
The range of value	< 3.2	3.2–6.3	6.3–10.7	10.7–17.8	> 17.8					
Major cardiovascular events	3.6	3.5	2.5	2.9	5.9	1.41	1.02	1.14	1.74	0.007
	(2.8–4.3)	(2.8–4.3)	(1.9–3.1)	(2.3–3.6)	(4.9–6.9)	(1.03–1.92)	(0.72–1.44)	(0.81–1.61)	(1.27–2.36)	
Myocardial infarction	1.1	1.5	1.1	1.5	2.6	1.74	1.32	1.67	2.03	0.0168
	(0.6–1.5)	(1.0–2.0)	(0.7–1.5)	(1.1–2.0)	(1.9–3.3)	(1.02–2.97)	(0.75–2.30)	(0.99–2.83)	(1.23–3.37)	
Stroke	2.7	2.3	1.5	1.7	3.8	1.28	0.9	0.94	1.64	0.0732
	(2.0–3.3)	(1.7–2.8)	(1.0–1.9)	(1.2–2.2)	(3.0–4.6)	(0.88–1.87)	(0.58–1.39)	(0.61–1.46)	(1.12–2.41)	

Hazard ratios and 95% CIs have been adjusted for age, gender, urban or rural location, education level, income, physical activity, smoking status, alcohol intake, BMI, history of hypertension, diabetes, total intake of energy, SFA, grains, vegetables, and dietary fiber. The Cox frailty model was used with household identification as random intercepts. Major cardiovascular events include myocardial infarction and stroke.

**Figure 2:**
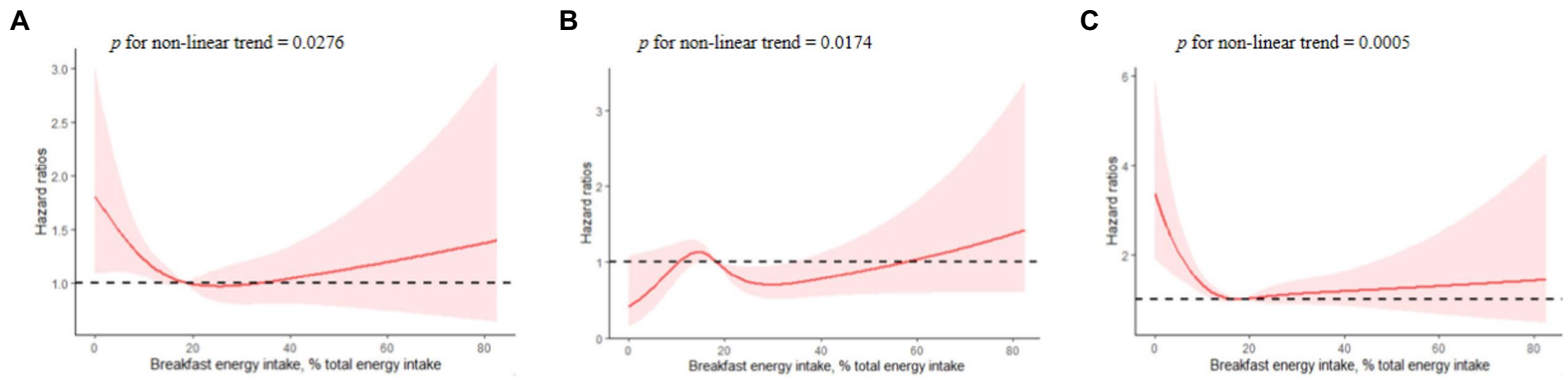
Multivariable-adjusted hazard ratios of association between breakfast energy intake and cardiovascular events [**(A)** major cardiovascular events; **(B)** myocardial infarction; **(C)** new-onset stroke]. A knot is located at the 5th, 35th, 65th, and 95^th^ percentiles for energy intake at breakfast. The model was fully adjusted for age, gender, urban or rural location, education level, income, physical activity, smoking status, alcohol intake, BMI, history of hypertension, diabetes, total intake of energy, SFA, grains, vegetables, and dietary fiber, with household identification as random intercepts.

For breakfast energy intake, we observed a possible non-linear relationship between breakfast energy intake and cardiovascular outcomes. In Figure 2, we used a restricted cubic spline to visualize the relationship between the risk of CVD and energy intake at breakfast on a continuous scale. Multivariable-adjusted restricted cubic spline further showed a significant L-shaped association of breakfast energy intake with stroke (Figure 2C). The L-shaped curve indicated that energy intake at breakfast was inversely associated with the risk of stroke at lower intake levels, but higher intakes increased the risk when exceeding a certain threshold. That is to say, breakfast energy intake that is too low or too high might increase the risk of stroke. Correspondingly, the relationship between energy intake at breakfast and major cardiovascular events was mainly U-shaped (Figure 2A), given that the first half of the curve was relatively flat. The U-shaped curve indicated that energy intake at breakfast that is too low or too high might also increase the risk of major cardiovascular events.

In the analysis for energy and major nutrients from breakfast, we evaluated the associations of intakes of energy, carbohydrates, protein, and fat at breakfast with risks of major cardiovascular events, MI, and stroke by sex (Supplementary Table 1). For male participants, adequate intake of energy at breakfast may reduce the risk of stroke (quintile 5 vs. quintile 1, HR 0.56 [95%CI 0.36–0.88]; *p*_trend_ = 0.0419). For female participants, higher percentage energy from carbohydrate intake at breakfast was associated with lower risk of major cardiovascular events (quintile 5 vs. quintile 1, HR 0.47 [95%CI 0.30–0.74]; *p*_trend_ = 0.0008), stroke (quintile 5 vs. quintile 1, HR 0.48 [95%CI 0.26–0.88]; *p*_trend_ = 0.0006). Percentage energy from protein intake at breakfast was associated with higher risk of myocardial infarction (quintile 5 vs. quintile 1, HR 2.49 [95%CI 1.21–5.11]; *p*_trend_ = 0.2641). In addition, breakfast proteins were divided into animal and plant proteins and then compared separately by sex with regard to health outcomes, but no association was found (Supplementary Table 2).

### 3.3. Substitution of carbohydrate intake with protein and fat intake at breakfast and cardiovascular risk

**Figure 3:**
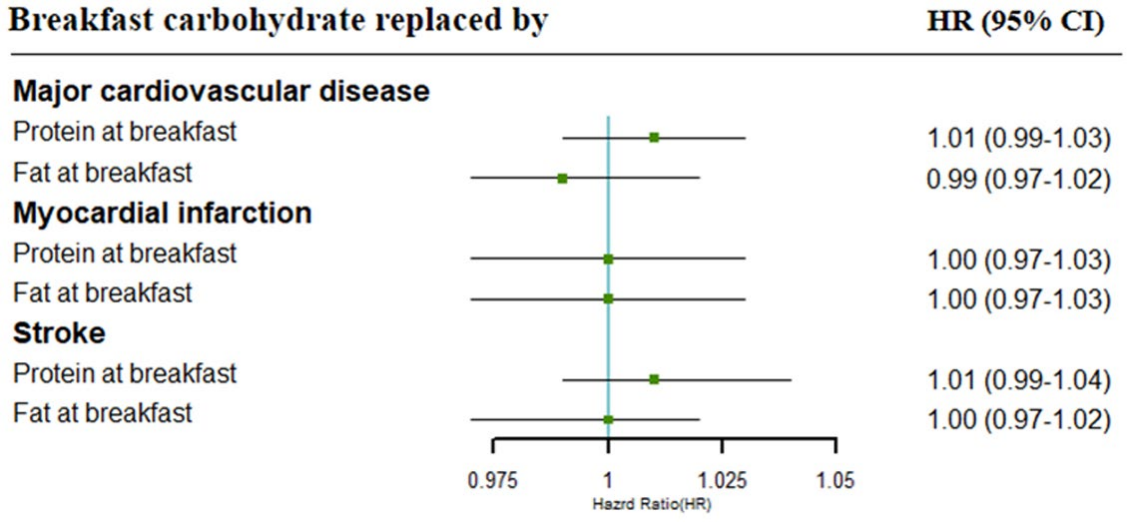
Cardiovascular risk associated with isocaloric (5% of energy) replacement of carbohydrates with other specific macronutrients. Adjustments included age, gender, urban or rural location, education level, income, physical activity, smoking status, alcohol intake, BMI, history of hypertension, diabetes, energy intake at breakfast, SFA, grains, vegetables, and dietary fiber.

No significant associations with the risk of major cardiovascular disease, MI, and stroke were found for the replacement of carbohydrates at breakfast with protein or fat at breakfast (Figure 3).

### 3.4. Associations with energy and macronutrient intake and health outcomes according to the presence of hypertension

**Figure 4:**
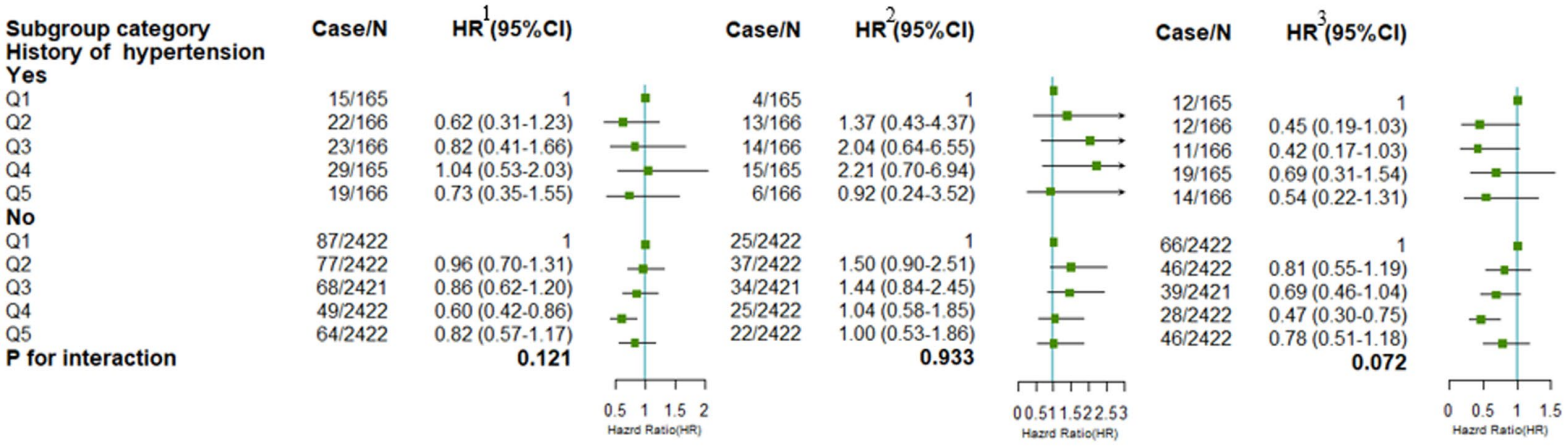
Stratified analyses by the history of hypertension at baseline of the association between breakfast energy intake and health outcomes. Adjustments included age, gender, urban or rural location, education level, income, physical activity, smoking status, alcohol intake, BMI, history of diabetes, total intake of energy, SFA, grains, vegetables, and dietary fiber, if not stratified. ^1^ refers to major cardiovascular events; ^2^ refers to myocardial infarction; ^3^ refers to new-onset stroke.

We further examined associations with energy and macronutrient intake and health outcomes according to the presence of hypertension at baseline. There were no significant interactions between breakfast energy intake and cardiovascular events (Figure 4). However, significant interactions were tested between macronutrient intake and a history of hypertension. We conducted stratification analyses to investigate the effect of percentage energy from carbohydrate, protein, and fat intake at breakfast on health outcomes. The higher percentage of energy from carbohydrate intake was associated with a lower risk of major cardiovascular events and new-onset stroke in participants without the presence of hypertension at baseline (Supplementary Figure 1). On the contrary, a percentage of energy from protein or fat intake was positively associated with risks of major cardiovascular events and new-onset stroke in participants without hypertension (Supplementary Figures 2, 3).

### 3.5. Sensitivity analysis

After additional adjustment for skipped breakfast, sensitivity analyses showed that the relationship among major cardiovascular events, myocardial infarction, new-onset stroke, and macronutrient intake remained consistent with the previous results, with a more significant *p*_trend_ (Supplementary Table 3).

## 4. Discussion

Our study demonstrated that, based on this nationally representative sample of Chinese adults, adequate energy intake at breakfast might be associated with major cardiovascular events and stroke. Second, we also found that a relatively higher intake of carbohydrates at breakfast was associated with a lower risk of cardiovascular disease. By contrast, a higher intake of protein and fat at breakfast was positively associated with the risk of cardiovascular disease. The link between the proportion of breakfast carbohydrates, protein, and fat intake and reduced cardiovascular events was stronger in participants without hypertension.

Previous studies have focused on the relationship between breakfast consumption and cardiovascular and metabolic disorders. The higher the breakfast energy intake, the lower the risk of CVD, as demonstrated in Western countries (10, 17). We also found that more energy intakes at breakfast may reduce the risk of stroke in men, which was consistent with the CIRCS study in the Japanese population (23). An animal experiment showed that high energy intake at breakfast reversed the expression of disrupted clock genes and had beneficial effects on lipid control (44). This conclusion is largely based on selective subgroup comparisons between skipping/irregular breakfast and regular breakfast eating, despite a recent finding that the benefits of breakfast intake may be related to meal quality rather than skipping/eating breakfast, due to unknown confounding factors (14). In the current study, an important finding was that appropriate intake of energy at breakfast was positively associated with the risk of major CVD and stroke, and the associations were independent of skipping breakfast. But there was no significant relationship of breakfast energy intake with MI. In conducting independent analyses of breakfast energy intake and MI, the effect of stroke as a factor may have been overlooked as to a few samples that experienced a stroke before MI. This may be due to changes in lifestyle and eating habits following a stroke, which in turn affects the relationship between breakfast energy intake and MI.

As a source of carbohydrates in the body, carbohydrates are one of the essential energy-yielding nutrients for the human body to sustain life activities. The “Dietary Guidelines for Chinese Residents (2022)” recommends that the energy provided by carbohydrates in the daily diet account for 50 to 60% of the total energy, but does not mention the proportion of energy intake at breakfast. In this study, we concluded that energy from carbohydrates at breakfast accounted for 76.4% of breakfast energy intake, which was similar to what was found in a previous study (45). The “carbohydrate-insulin model” has been proposed, arguing that a high intake of carbohydrates leads to endocrine dysregulation marked by hyperinsulinemia, driving energy allocation and increased energy storage in adipose tissue (46, 47). Although high intake of carbohydrates was linked to cardiovascular risk factors, it was unknown whether carbohydrate intake at breakfast affects the body. Our research revealed a very interesting conclusion that a higher intake of breakfast carbohydrates was associated with a reduced risk of CVD, especially in female population, which was contrary to other studies (48–50). One reason is possibly that the energy provided by breakfast carbohydrate intake is derived from the calculation of breakfast energy intake in our study rather than total daily energy intake in other studies. This suggests that a higher intake of carbohydrates, compared to protein and fat at breakfast, was beneficial for preventing cardiovascular events. Interestingly, a systematic review has indicated that carbohydrate quality may be more important than quantity when assessing the relationship between carbohydrate intake and cardiometabolic outcomes (51). Therefore, future studies should further evaluate the association between food sources of breakfast carbohydrates and CVD.

In the current study, there were no associations between source-specific protein intake (e.g., animal versus plant sources) and a range of CVD risk. Breakfast protein intake was positively associated with CVD incidence, which was inconsistent with many previous studies (52, 53). By the comparison of two systematic reviews (26, 54), we found differences in the associations between the exposure variables of meat as a food group and protein as a nutrient and CVD. In addition, to the best of our knowledge, dietary patterns have been undergoing rapid transitions in China over the past two decades. The amount of consuming meat continued to increase dramatically, with livestock and poultry intake far above the recommendations (55). Moreover, adults eating away-from-home meals are more likely to consume processed meat, which may contribute to the burden of CVD. Considering this may be the reason for the inconsistent results of our study and other studies based on the non-Chinese population.

For breakfast fat intake, we found a positive correlation between energy intake from fat and the risk of major cardiovascular events, myocardial infarction, and stroke. Previous studies have demonstrated the effect of breakfast fat intake on cardiovascular health. Granulocyte colony-stimulating factor (G-CSF) and granulocyte-monocyte colony-stimulating factor (GM-CSF) were elevated after a high-fat breakfast, which has implications for adipose tissue inflammation and the risk of developing atherosclerosis (56). Additionally, a crossover clinical trial has suggested that dietary intake of unsaturated fatty acids at breakfast may induce an anti-inflammatory response, in turn affecting cardiometabolic risk (57). Inconsistent with our findings, the replacement of saturated fatty acids with unsaturated fatty acids may reduce cardiovascular risk (21, 22). However, a recent systematic review showed a lack of rigorous evidence for limiting the consumption of saturated fatty acids or replacing saturated fatty acids with polyunsaturated fatty acids (58). There is much evidence that eating a high-fat breakfast has adverse effects on cardiovascular health, but further well-conducted cohort studies are needed to identify the relationship between types of fatty acids and cardiovascular health.

To our knowledge, this is the first longitudinal study to use a health and nutrition survey database in a large sample population from nine provinces in China. It is noteworthy that our study yielded somewhat different results from previous studies by a risk assessment of the association between breakfast energy and macronutrient intake and cardiovascular disease. However, our study had some limitations. First, self-reported disease diagnosis was subjective and potentially associated with recall bias. Second, although the adjustment for socioeconomic and lifestyle factors and intakes of main food groups, unknown confounding factors that we did not account for are possible. Third, the 24-h meal recall method generally cannot accurately assess daily dietary intake.

## 5. Conclusion

Breakfast is part of the daily diet, and eating breakfast every day is also a healthy lifestyle advocated by the World Health Organization. Chinese dietary nutritional guidelines also point out that a reasonable diet, part of which is eating breakfast regularly, can reduce the risk of cardiovascular disease. As the first meal of the day, breakfast is very important for dietary nutrition intake. In conclusion, a higher intake of carbohydrates, and a relatively lower intake of protein and fat at breakfast may contribute to cardiovascular health on the basis of maintaining a moderate energy intake. This information is of importance in providing nutritional recommendations, especially a reasonable combination of breakfast nutrition for the public. Therefore, in the perspective of the future, we need to further explore the impact of macronutrient quality on cardiovascular disease in addition to macronutrient quantity.

## Data availability statement

The datasets presented in this study can be found in online repositories. The names of the repository/repositories and accession number (s) can be found in the article/Supplementary material.

## Ethics statement

The studies involving human participants were reviewed and approved by the Institutional Review Committees of the University of North Carolina at Chapel Hill and the National Institute of Nutrition and Food Safety, Chinese Center for Disease Control and Prevention. The patients/participants provided their written informed consent to participate in this study.

## Author contributions

XD and RY made substantial contributions to the conception and design of the study. XD was a major contributor in writing the first draft of the manuscript. Data collection and statistical analysis were performed by MM and SK with assistance from XD. RY, JZ, and XT reviewed and commented on the first draft. All authors contributed to the article and approved the submitted version.

## Conflict of interest

The authors declare that the research was conducted in the absence of any commercial or financial relationships that could be construed as a potential conflict of interest.

## Publisher’s note

All claims expressed in this article are solely those of the authors and do not necessarily represent those of their affiliated organizations, or those of the publisher, the editors and the reviewers. Any product that may be evaluated in this article, or claim that may be made by its manufacturer, is not guaranteed or endorsed by the publisher.

## Abbreviations

CVD: cardiovascular disease
MI: myocardial infarction
SFA: saturated fatty acid
SBP: systolic blood pressure
DBP: diastolic blood pressure
BMI: body mass index
MET: metabolic equivalent of task
CHNS: China health and nutrition survey
SD: stand deviations
CI: confidence interval
HR: hazard ratios
