# Supplementary material for: The association of energy and macronutrient intake at breakfast and cardiovascular disease in Chinese adults: From a 14-year follow-up cohort study
Source: Front Nutr. 2023 Mar 20;10:1093561. doi: 10.3389/fnut.2023.1093561 (PMC10069285; doi:10.3389/fnut.2023.1093561)
Supplement: Supplementary file 1 [file Data_Sheet_1.docx]

**Supplementary Table 1. Association between energy and percentage energy from macronutrients at breakfast and health outcomes by sex.**

|  | Quintile 1 | Quintile 2 | Quintile 3 | Quintile 4 | Quintile 5 | P _trend_ |
| --- | --- | --- | --- | --- | --- | --- |
| **Men (N=6286)** |  |  |  |  |  |  |
| **Energy at breakfast (kcal/d)** |  |  |  |  |  |  |
| Major cardiovascular events | 1.00(ref) | 0.80(0.55, 1.19) | 0.70(0.47, 1.05) | 0.67(0.45, 1.00) | 0.64(0.42, 0.96) | 0.093 |
| Myocardial infarction | 1.00(ref) | 2.59(1.18, 5.70) | 2.69(1.21, 5.99) | 2.24(1.00, 5.10) | 1.50(0.62, 3.62) | 0.3453 |
| Stroke | 1.00(ref) | 0.58(0.37, 0.91) | 0.48(0.30, 0.79) | 0.51(0.33, 0.80) | 0.56(0.36, 0.88) | 0.0419 |
| **Energy from carbohydrate (% breakfast energy)** |  |  |  |  |  |  |
| Major cardiovascular events | 1.00(ref) | 0.72(0.49, 1.06) | 0.62(0.41, 0.94) | 0.65(0.44, 0.97) | 0.75(0.50, 1.13) | 0.0026 |
| Myocardial infarction | 1.00(ref) | 1.16(0.61, 2.21) | 0.91(0.46, 1.79) | 0.68(0.33, 1.40) | 0.79(0.39, 1.63) | 0.6033 |
| Stroke | 1.00(ref) | 0.59(0.37, 0.94) | 0.52(0.32, 0.84) | 0.66(0.42, 1.03) | 0.72(0.46, 1.15) | 0.0015 |
| **Energy from protein (% breakfast energy)** |  |  |  |  |  |  |
| Major cardiovascular events | 1.00(ref) | 1.17(0.78, 1.76) | 0.96(0.63, 1.46) | 0.88(0.57, 1.36) | 1.47(0.99, 2.19) | 0.0003 |
| Myocardial infarction | 1.00(ref) | 1.00(0.48, 2.10) | 1.09(0.53, 2.24) | 1.32(0.65, 2.65) | 1.24(0.61, 2.54) | 0.2469 |
| Stroke | 1.00(ref) | 1.23(0.77, 1.95) | 0.88(0.54, 1.44) | 0.69(0.41, 1.17) | 1.54(0.98, 2.40) | 0.0002 |
| **Energy from fat (% breakfast energy)** |  |  |  |  |  |  |
| Major cardiovascular events | 1.00(ref) | 1.21(0.83, 1.75) | 0.76(0.49, 1.18) | 0.77(0.50, 1.19) | 1.45(0.98, 2.13) | 0.0028 |
| Myocardial infarction | 1.00(ref) | 2.35(1.00, 5.50) | 1.94(0.80, 4.69) | 1.85(0.75, 4.56) | 3.37(1.46, 7.79) | 0.0364 |
| Stroke | 1.00(ref) | 1.05(0.69, 1.59) | 0.56(0.34, 0.94) | 0.67(0.41, 1.09) | 1.20(0.78, 1.87) | 0.0145 |
| **Women (N=6651)** |  |  |  |  |  |  |
| **Energy at breakfast (kcal/d)** |  |  |  |  |  |  |
| Major cardiovascular events | 1.00(ref) | 0.89(0.61, 1.31) | 0.91(0.60, 1.37) | 0.49(0.28, 0.86) | 0.97(0.58, 1.61) | 0.9168 |
| Myocardial infarction | 1.00(ref) | 1.14(0.68, 1.91) | 0.98(0.56, 1.71) | 0.55(0.27, 1.12) | 0.81(0.39, 1.66) | 0.4068 |
| Stroke | 1.00(ref) | 0.67(0.39, 1.15) | 0.80(0.46, 1.39) | 0.39(0.17, 0.90) | 0.87(0.44, 1.73) | 0.7964 |
| **Energy from carbohydrate (% breakfast energy)** |  |  |  |  |  |  |
| Major cardiovascular events | 1.00(ref) | 0.51(0.33, 0.79) | 0.60(0.39, 0.92) | 0.43(0.27, 0.67) | 0.47(0.30, 0.74) | 0.0008 |
| Myocardial infarction | 1.00(ref) | 0.69(0.39, 1.21) | 0.71(0.41, 1.23) | 0.51(0.27, 0.94) | 0.58(0.31, 1.08) | 0.1367 |
| Stroke | 1.00(ref) | 0.36(0.18, 0.71) | 0.54(0.30, 0.97) | 0.39(0.21, 0.73) | 0.48(0.26, 0.88) | 0.0006 |
| **Energy from protein (% breakfast energy)** |  |  |  |  |  |  |
| Major cardiovascular events | 1.00(ref) | 1.02(0.61, 1.69) | 1.23(0.75, 2.01) | 1.31(0.81, 2,11) | 1.77(1.12, 2.79) | 0.1162 |
| Myocardial infarction | 1.00(ref) | 1.33(0.61, 2.91) | 1.47(0.69, 3.12) | 2.30(1.14, 4.62) | 2.49(1.21, 5.11) | 0.2641 |
| Stroke | 1.00(ref) | 0.76(0.40, 1.43) | 0.99(0.54, 1.83) | 0.68(0.36, 1.31) | 1.13(0.64, 1.99) | 0.3869 |
| **Energy from fat (% breakfast energy)** |  |  |  |  |  |  |
| Major cardiovascular events | 1.00(ref) | 0.88(0.55, 1.39) | 0.82(0.51, 1.33) | 1.05(0.66, 1.66) | 1.47(0.95, 2.30) | 0.0045 |
| Myocardial infarction  Stroke | 1.00(ref)  1.00(ref) | 1.08(0.57, 2.05)  0.68(0.36, 1.29) | 0.83(0.42, 1.65)  0.78(0.42, 1.46) | 1.36(0.74, 2.51)  0.67(0.33, 1.33) | 1.37(0.72, 2.61)  1.39(0.77, 2.51) | 0.1355  0.0068 |

Hazard ratios and 95% CIs have been adjusted for age, urban or rural location, education level, income, physical activity, smoking status, alcohol intake, BMI, history of hypertension, diabetes, total energy intake, SFA, grains, vegetables, and dietary fiber. Cox frailty model was used with household identification as random intercepts. Major cardiovascular events include myocardial infarction and stroke.

**Supplementary Table 2. Association between animal protein, plant protein at breakfast and health outcomes by sex.**

|  | Quintile 1 | Quintile 2 | Quintile 3 | Quintile 4 | Quintile 5 | P _trend_ |
| --- | --- | --- | --- | --- | --- | --- |
| **All participants (N=12937)** |  |  |  |  |  |  |
| **Energy from animal protein (% breakfast energy)** |  |  |  |  |  |  |
| Major cardiovascular events | 1.00(ref) | 0.89(0.66, 1.21) | 0.87(0.66, 1.14) | 0.81(0.62, 1.07) | 0.79(0.59, 1.05) | 0.7646 |
| Myocardial infarction | 1.00(ref) | 0.79(0.49, 1.28) | 1.02(0.69, 1.52) | 0.87(0.57, 1.31) | 0.84(0.55, 1.31) | 0.3276 |
| Stroke | 1.00(ref) | 0.98(0.68, 1.41) | 0.75(0.53, 1.06) | 0.76(0.54, 1.06) | 0.78(0.54, 1.12) | 0.8795 |
| **Energy from plant protein (% breakfast energy)** |  |  |  |  |  |  |
| Major cardiovascular events | 1.00(ref) | 1.28(0.98, 1.69) | 0.77(0.56, 1.05) | 1.00(0.73, 1.37) | 0.91 (0.66, 1.25) | 0.8493 |
| Myocardial infarction | 1.00(ref) | 1.25(0.82, 1.93) | 0.81(0.51, 1.30) | 1.02(0.64, 1.62) | 0.89(0.55, 1.43) | 0.6226 |
| Stroke | 1.00(ref) | 1.23(0.88, 1.72) | 0.67(0.45, 0.99) | 0.99(0.67, 1.47) | 0.89(0.59, 1.32) | 0.6720 |
| **Men (N=6286)** |  |  |  |  |  |  |
| **Energy from animal protein (% breakfast energy)** |  |  |  |  |  |  |
| Major cardiovascular events | 1.00(ref) | 0.96(0.64, 1.45) | 0.82(0.55, 1.20) | 0.86(0.60, 1.24) | 0.92(0.64, 1.33) | 0.5159 |
| Myocardial infarction | 1.00(ref) | 1.47(0.75, 2.87) | 1.03(0.53, 2.01) | 0.84(0.42, 1.67) | 1.34(0.73, 2.47) | 0.9883 |
| Stroke | 1.00(ref) | 0.88(0.55, 1.40) | 0.73(0.47, 1.13) | 0.82(0.54, 1.22) | 0.75(0.48, 1.16) | 0.6082 |
| **Energy from plant protein (% breakfast energy)** |  |  |  |  |  |  |
| Major cardiovascular events | 1.00(ref) | 1.22(0.86, 1.75) | 0.75(0.50, 1.14) | 0.89(0.59, 1.34) | 0.81(0.53, 1.24) | 0.9471 |
| Myocardial infarction | 1.00(ref) | 0.94(0.51, 1.73) | 0.58(0.28, 1.19) | 0.79(0.41, 1.52) | 0.50(0.23, 1.05) | 0.6605 |
| Stroke | 1.00(ref) | 1.22(0.81, 1.83) | 0.72(0.44, 1.17) | 0.83(0.51, 1.36) | 0.86(0.53, 1.39) | 0.9629 |
| **Women (N=6651)** |  |  |  |  |  |  |
| **Energy from animal protein (% breakfast energy)** |  |  |  |  |  |  |
| Major cardiovascular events | 1.00(ref) | 0.82(0.52, 1.28) | 0.95(0.65, 1.41) | 0.76(0.50, 1.15) | 0.63(0.39, 1.01) | 0.0528 |
| Myocardial infarction | 1.00(ref) | 0.45(0.22, 0.94) | 0.98(0.60, 1.62) | 0.87(0.51, 1.46) | 0.52(0.27, 1.02) | 0.0847 |
| Stroke | 1.00(ref) | 1.18(0.68, 2.06) | 0.86(0.48, 1.51) | 0.67(0.35, 1.27) | 0.89(0.49, 1.64) | 0.3216 |
| **Energy from plant protein (% breakfast energy)** |  |  |  |  |  |  |
| Major cardiovascular events | 1.00(ref) | 1.44(0.94, 2.22) | 0.84(0.51, 1.36) | 1.19(0.73, 1.93) | 1.05(0.64, 1.72) | 0.9414 |
| Myocardial infarction | 1.00(ref) | 1.74(0.93, 3.26) | 1.11(0.57, 2.16) | 1.31(0.66, 2.57) | 1.39(0.72, 2.69) | 0.2726 |
| Stroke | 1.00(ref) | 1.34(0.75, 2.37) | 0.63(0.31, 1.26) | 1.41(0.75, 2.67) | 0.96(0.48, 1.92) | 0.4296 |

Hazard ratios and 95% CIs have been adjusted for age, urban or rural location, education level, income, physical activity, smoking status, alcohol intake, BMI, history of hypertension, diabetes, total energy intake, SFA, grains, vegetables, meat and dietary fiber. Cox frailty model was used with household identification as random intercepts. Major cardiovascular events include myocardial infarction and stroke.

**Supplementary Table 3. Sensitive analysis by addition of breakfast skipping of association between energy and macronutrient intake and health outcomes.**

|  | Quintile 1 | Quintile 2 | Quintile 3 | Quintile 4 | Quintile 5 | P _trend_ |
| --- | --- | --- | --- | --- | --- | --- |
| **Energy at breakfast (kcal/d)** |  |  |  |  |  |  |
| Major cardiovascular events | 1.00 (ref) | 0.93 (0.70-1.25) | 0.89 (0.66-1.21) | 0.70 (0.51-0.97) | 0.85 (0.60-1.19) | 0.1221 |
| Myocardial infarction | 1.00 (ref) | 1.45 (0.93-2.28) | 1.39 (0.88-2.19) | 0.99 (0.61-1.62) | 0.91 (0.53-1.59) | 0.384 |
| Stroke | 1.00 (ref) | 0.72 (0.50-1.03) | 0.72 (0.49-1.05) | 0.60 (0.40-0.90) | 0.80 (0.53-1.20) | 0.1739 |
| **Energy from carbohydrate (% breakfast energy)** |  |  |  |  |  |  |
| Major cardiovascular events | 1.00 (ref) | 0.67 (0.49-0.90) | 0.70 (0.51-0.95) | 0.58 (0.43-0.79) | 0.56 (0.41-0.78) | 0.0005 |
| Myocardial infarction | 1.00 (ref) | 0.86 (0.56-1.32) | 0.82 (0.53-1.28) | 0.60 (0.37-0.98) | 0.64 (0.39-1.03) | 0.0272 |
| Stroke | 1.00 (ref) | 0.57 (0.38-0.84) | 0.63 (0.43-0.94) | 0.60 (0.41-0.86) | 0.56 (0.37-0.84) | 0.0126 |
| **Energy from protein (% breakfast energy)** |  |  |  |  |  |  |
| Major cardiovascular events | 1.00 (ref) | 1.36 (0.95-1.94) | 1.27 (0.88-1.83) | 1.32 (0.91-1.92) | 1.86 (1.30-2.65) | 0.0015 |
| Myocardial infarction | 1.00 (ref) | 1.18 (0.68-2.08) | 1.27 (0.73-2.20) | 1.78 (1.04-3.03) | 1.77 (1.03-3.04) | 0.0091 |
| Stroke | 1.00 (ref) | 1.35 (0.88-2.08) | 1.17 (0.75-1.82) | 0.96 (0.60-1.54) | 1.68 (1.09-2.61) | 0.1081 |
| **Energy from fat (% breakfast energy)** |  |  |  |  |  |  |
| Major cardiovascular events | 1.00 (ref) | 1.48 (1.06-2.07) | 1.07 (0.74-1.55) | 1.20 (0.84-1.74) | 1.84 (1.31-2.58) | 0.0054 |
| Myocardial infarction | 1.00 (ref) | 1.73 (0.99-3.02) | 1.31 (0.73-2.35) | 1.66 (0.96-2.90) | 2.02 (1.18-3.46) | 0.0282 |
| Stroke | 1.00 (ref) | 1.37 (0.91-2.07) | 0.96 (0.61-1.53) | 1.01 (0.64-1.61) | 1.78 (1.16-2.71) | 0.0467 |

Hazard ratios and 95% CIs are adjusted for age, gender, living area, education level, income, physical activity, smoking status, alcohol intake, BMI, hypertension, diabetes, total energy intake, SFA, grains, vegetables, dietary fiber, and breakfast skipping (Yes/No). The Cox frailty model was used with household identification as random intercepts. Major cardiovascular events include myocardial infarction and stroke.


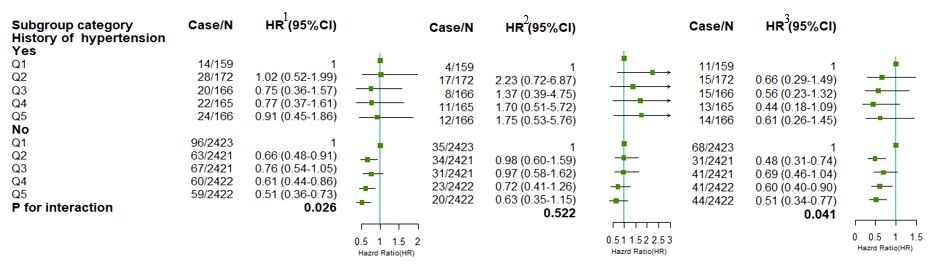


**Supplementary Figure 1.** Stratified analyses by the history of hypertension at baseline of the association between percentage energy from carbohydrates at breakfast and health outcomes. Adjustments included age, gender, living area, education level, income, physical activity, smoking status, alcohol intake, BMI, diabetes, total energy intake, SFA, grains, vegetables, and dietary fiber, if not stratified.


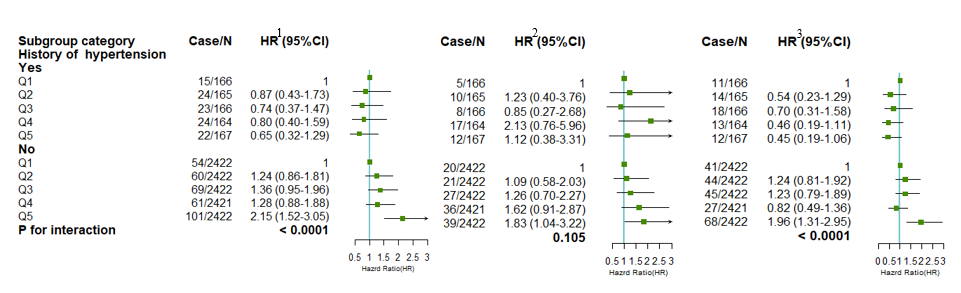


**Supplementary Figure 2.** Stratified analyses by the history of hypertension at baseline of the association between percentage energy from protein at breakfast and health outcomes. Adjustments included age, gender, living area, education level, income, physical activity, smoking status, alcohol intake, BMI, diabetes, total energy intake, SFA, grains, vegetables, and dietary fiber, if not stratified.


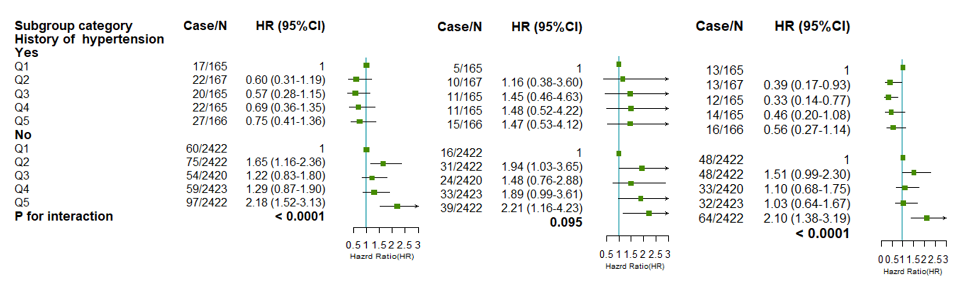


**Supplementary Figure 3.** Stratified analyses by the history of hypertension at baseline of the association between percentage energy from fat at breakfast and health outcomes. Adjustments included age, gender, living area, education level, income, physical activity, smoking status, alcohol intake, BMI, diabetes, total energy intake, SFA, grains, vegetables, and dietary fiber, if not stratified.
